# Actinomycosis and U. S. Meat Inspection

**Published:** 1892-03

**Authors:** W. L. Williams


### ACTINOMYCOSIS AND U. S. MEAT INSPECTION.

By W. L. Williams, V. S.

In the Journal for February we find an interesting and timely article on Actinomycosis from the pen of Dr. R. W. Hickman, U. S. Veterinary Inspector at Chicago, evidently suggested by the Greenhut–Pearson case, recently tried at Peoria, Ill., and in which Dr. Hickman appeared as a leading witness.

On the whole, the article is certainly quite instructive, and will well repay a careful perusal, although it seems that certain "malcontents and soreheads" have so wrought upon the Doctor's fine feelings as to have aroused a certain degree of resentment and an overwhelming desire for self-defense and glorification. He apparently assumes that a bureau to which he has been attached, through sheer force of competency, "and in honor above suspicion or reproach," should not be criticised.

There are countries in the world where criticisms of such a government bureau would not be tolerated, but under the Stars and Stripes it is quite allowable. Few government bureaus of equal importance, however, have received less unfriendly criticism than that to which Dr. Hickman has the honor to belong, and that special department in which he is engaged—meat inspection—has not, to our knowledge, received so far an unfavorable comment from the veterinary journals, and so we fail to comprehend his last sentence in which he refers to "contemptible articles that have appeared in our representative journals," etc.

In fact, the profession is a unit in favor of rigid meat inspection, and if there be those who doubt either the efficiency or honor of Dr. Salmon, they constitute certainly a very small minority.

The acts of certain individuals may have been criticised, as is perfectly legitimate, but this can not be deemed a criticism of the bureau, except it be an official act of the chief or at his direct command.

In so far as actinomycosis in its relation to meat inspection is concerned, no criticisms have been made of the bureau, because so far as is known it has taken no definite stand, so it is not open to criticism.

Dr. Hickman has, however, taken a definite stand in the matter in his article in February Journal, as well as in his testimony in the Greenhut-Pearson trial, which, by our laws and usages, is subject to criticism.

In his annual report dated 27th October, 1891, on page 14, the honorable Secretary of Agriculture says: "In most, if not all, European countries, inspectors, according to their reports, freely pass for consumption the meat of animals affected with foot-and-mouth disease, localized tuberculosis, actinomycosis and similar diseases, which, according to the views and customs of this country, must be condemned. But all the meat for the foreign market is inspected the same as that for home consumption.

A few weeks later in the trial of Peoria, Dr. Hickman deposed that "I want to express the idea that *I would not reject it* (carcass of actinomycotic animal) *simply because it had actinomycosis*, but I would reject an animal as unfit for food on general grounds, whether from actinomycotic tumor or any other tumor that had drained the system," and in reply to the question, "Would you consider a fat animal with a tumor that was discharging pus as fit for human food?" said: "If the animal was in a thrifty condition—yes sir." Then on page 114 of the JOURNAL he says: "Animals suffering from supperating actinomycotic or other tumors should be condemned on general grounds, and also on scientific principles, and not simply for sentimental reasons.

We trust Dr. Hickman will not suspect that we are criticising the bureau, when we suggest that the above quoted statements of Secretary Rusk and those of his own are diametrically opposed to each other, when the former officially states that actinomycotic carcasses are *condemned*, while Dr. Hickman solemnly swears that he *does not* condemn them, except when from great discharge of pus the animal system is drained, and then *not for actinomycosis*, but "on *general* grounds and also on scientific principles." The doctor would be doing us a great favor by explaining what he means by "general grounds" and "scientific principles."

We would be pleased to know what general grounds or scientific principles in meat inspection warrants the rejection of an animal bearing an open discharging abscess, and the passing of an animal with multiple unruptured abscesses. We would like to know how the meat of an animal is rendered dangerous to human consumers by an abscess having broken and discharged its contents, while it is rendered quite safe by the presence of one or many pus-containing abscesses. According to Dr. Hickman's theory, except he presses into service a new arm to his "scientific principles," he would pass as healthy meat, pork containing multiple actinomycotic abscesses.

By reading Dr. Hickman's paper on page 114 of the JOURNAL, one would be led to infer that Dr. Hickman had made an extensive study of the disease, and he lays special emphasis upon the fact that he has been furnished with one of the renowned Zeiss microscopes with all the latest attachments, including a microtome, Dr. Norgaard and an emersion lens.

In referring to the number of microscopic examinations in the Peoria trial, Dr. Hickman was asked: "That makes four (you have made) in all during your life?" A.—"Of different animals, yes, sir." At this rate of use the present outfit of appliances, if well cared for, should last for some time.

After stating at the Peoria trial that he had commenced his elaborate microscopical studies of actinomycosis during the summer of '91, and being asked if subsequent to that date he had regularly examined microscopically animals suspected to be affected with actinomycosis he answers: "Yes, sir; where we could get an opportunity *and get instruments of sufficient high power to see the germs.*"

We must consequently infer that the objectives with his fine Zeiss instrument are of too low power to see the germ, but are rather adapted for looking at larger objects, probably a live steer.

We regret very much that the inferences to be drawn from Dr. Hickman's statements in the JOURNAL are at such wide variance with his sworn testimony in the law courts, and trust he will be able to explain the discrepancies.

More serious, however, appears the want of harmony between the official declaration of the Secretary of Agriculture that all animals affected with actinomycosis are condemned for inter-state and international traffic, while Dr. Hickman swears that he *would not condemn an animal for actinomycosis*, although he admitted under oath that he would not pass live cattle so affected for export, but left the conclusion quite clear that after slaughter those parts which were evidently affected and would lead to the detection of the character of the meat were rejected, and the carcass thus robbed of its patent evidences of disease were passed as sound for inter-state and foreign trade.

He thus takes a diseased animal, decapitates it, and renders the remainder of the carcass sound.

If decapitation renders such a carcass sound, it would seem quite allowable to tag a live export steer "sound, except his head," and permit decapitation at the point of consumption.

